# Local calcium signal transmission in mycelial network exhibits decentralized stress responses

**DOI:** 10.1093/pnasnexus/pgad012

**Published:** 2023-03-07

**Authors:** Ayaka Itani, Shunsuke Masuo, Riho Yamamoto, Tomoko Serizawa, Yu Fukasawa, Naoki Takaya, Masatsugu Toyota, Shigeyuki Betsuyaku, Norio Takeshita

**Affiliations:** Microbiology Research Center for Sustainability (MiCS), Faculty of Life and Environmental Sciences, University of Tsukuba, Tennodai 1-1-1, Tsukuba, 305-8572, Japan; Microbiology Research Center for Sustainability (MiCS), Faculty of Life and Environmental Sciences, University of Tsukuba, Tennodai 1-1-1, Tsukuba, 305-8572, Japan; Microbiology Research Center for Sustainability (MiCS), Faculty of Life and Environmental Sciences, University of Tsukuba, Tennodai 1-1-1, Tsukuba, 305-8572, Japan; Microbiology Research Center for Sustainability (MiCS), Faculty of Life and Environmental Sciences, University of Tsukuba, Tennodai 1-1-1, Tsukuba, 305-8572, Japan; Graduate School of Agricultural Science, Tohoku University, 232-3 Yomogida, Naruko, Osaki, Miyagi, 989-6711, Japan; Microbiology Research Center for Sustainability (MiCS), Faculty of Life and Environmental Sciences, University of Tsukuba, Tennodai 1-1-1, Tsukuba, 305-8572, Japan; Department of Biochemistry and Molecular Biology, Saitama University, 255 Shimo-Okubo, Sakura-ku, Saitama, 338-8570, Japan; Suntory Rising Stars Encouragement Program in Life Sciences (SunRiSE), Kyoto, Japan; Department of Botany, University of Wisconsin-Madison, 430 Lincoln Drive, Madison, WI 53706, USA; Department of Plant Life Science, Faculty of Agriculture, Ryukoku University, 1-5 Yokotani, Seta Oe-cho, Otsu, Shiga, 520-2194Japan; Microbiology Research Center for Sustainability (MiCS), Faculty of Life and Environmental Sciences, University of Tsukuba, Tennodai 1-1-1, Tsukuba, 305-8572, Japan

**Keywords:** calcium signal, mycelia, calmodulin, calmodulin-dependent kinases, fungi

## Abstract

Many fungi live as mycelia, which are networks of hyphae. Mycelial networks are suited for the widespread distribution of nutrients and water. The logistical capabilities are critical for the extension of fungal survival areas, nutrient cycling in ecosystems, mycorrhizal symbioses, and virulence. In addition, signal transduction in mycelial networks is predicted to be vital for mycelial function and robustness. A lot of cell biological studies have elucidated protein and membrane trafficking and signal transduction in fungal hyphae; however, there are no reports visualizing signal transduction in mycelia. This paper, by using the fluorescent Ca^2+^ biosensor, visualized for the first time how calcium signaling is conducted inside the mycelial network in response to localized stimuli in the model fungus *Aspergillus nidulans*. The wavy propagation of the calcium signal inside the mycelium or the signal blinking in the hyphae varies depending on the type of stress and proximity to the stress. The signals, however, only extended around 1,500 μm, suggesting that the mycelium has a localized response. The mycelium showed growth delay only in the stressed areas. Local stress caused arrest and resumption of mycelial growth through reorganization of the actin cytoskeleton and membrane trafficking. To elucidate the downstream of calcium signaling, calmodulin, and calmodulin-dependent protein kinases, the principal intracellular Ca^2+^ receptors were immunoprecipitated and their downstream targets were identified by mass spectrometry analyses. Our data provide evidence that the mycelial network, which lacks a brain or nervous system, exhibits decentralized response through locally activated calcium signaling in response to local stress.

**Significance Statement**

The mycelial network of fungi sometimes extends several meters into the soil. The ability of mycelial networks to transport nutrients and water affects not only fungal growth but also plants and forest ecosystems. In addition to their logistical capabilities, signal transduction in mycelial networks, which lack a brain or nervous system, is vital for mycelial function and robustness and may also be for fungal memory or intelligence. This study reveals how information is transmitted within the mycelial network by visualizing calcium signals and identifying its downstream targets. Our data provide evidence that mycelia exhibit decentralized responses rather than centralized ones by causing local calcium signaling in response to local stress.

## Introduction

Most fungi form mycelia by repeatedly elongating and branching hyphae (1, 2). They secrete numerous degradative enzymes outside, degrade biopolymers in the environment, and get nutrition by absorbing them during mycelial development. Mycelia are well-suited for adsorption on nutritional substrate and entrance into them, which adds a great advantage to fungi in infection/decomposition of large and solid substrates (2–4). The network structure of mycelium allows for the widespread distribution of absorbed nutrients across the network. This logistical role is critical for the extension of fungal survival area, nutrient cycling in ecosystems, mycorrhizal symbioses, and virulence (5–7). Mycorrhizal networks connect neighboring plants in the soil and contribute to their nutritional distribution (8). In addition to nutrition logistics, signal transmission such as stress response is predicted to be crucial for the mycelial function and robustness (9), although little is known about signal transduction in mycelial networks.

Cells of multicellular organisms are integrated into a single living system through cellular communication. Calcium signaling, defined as a change in intracellular Ca^2+^ concentration, serves as a second messenger for transmitting biological information between cells in several species (10). Calcium signaling, for example, is required for chemical exchange and information transfer between neurons in neural networks (11). In another example, when plants are exposed to environmental stressors, such as drought or damage, calcium signaling is activated, which triggers downstream responses (12). When one leaf is damaged, calcium signal moves via the veins, which carry water and nutrients throughout the plant, to leaves away from the injured leaf and throughout the plant body (13). Calmodulin (CaM) and calcium/CaM-dependent protein kinase (CaMK) are the principal intracellular Ca^2+^ receptors and are responsible for conveying these signals to numerous target proteins (14). Ca^2+^-bound and activated CaM binds directly to target proteins, altering their activity. CaMK phosphorylates target proteins to regulate their activity.

Most Dikarya fungi form multicellular mycelia whose hyphae are segregated by septa, although the cytoplasm is continually linked because the septa have tiny pores (15). Recent research has shown that oscillatory Ca^2+^ inflow at a hyphal tip influences the timing of actin depolymerization and exocytosis, resulting in oscillatory hyphal growth (16, 17). This study investigated the conduction of calcium signaling within the mycelial network and each hypha in response to localized stimuli. Green fluorescent protein (GFP)-trap of CaM/CaMK and mass spectrometry analyses identified downstream targets of calcium signaling.

## Results

### Conduction of calcium signaling in mycelium by cutting stress

**Fig. 1.**
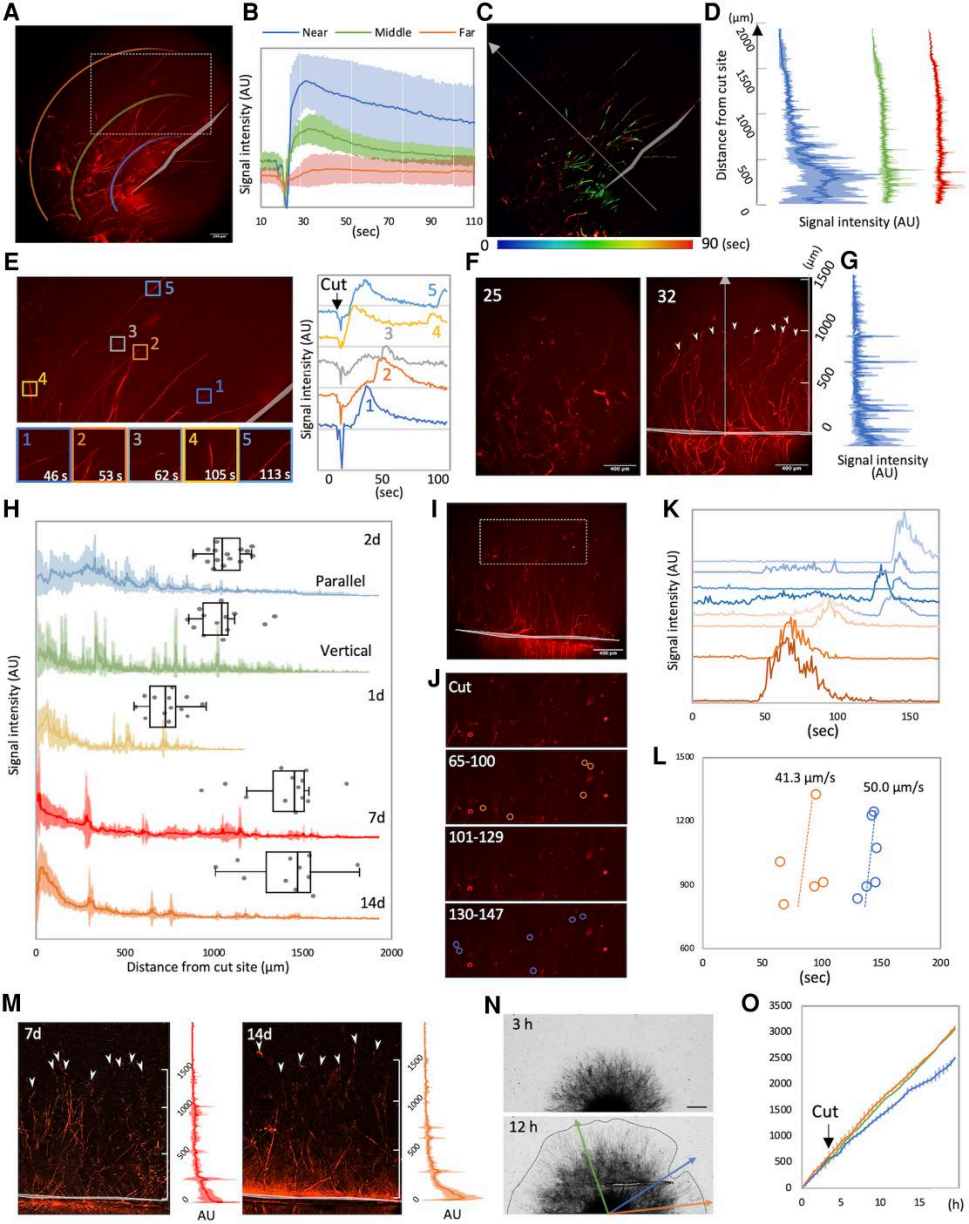
Conduction of calcium signal in the cut mycelia. A) Image of calcium signal immediately after cutting the mycelium, from Video 1. The cut site is indicated by a gray line. According to the distance from the cut site, the regions were classified as near, middle and far. B) Time course of signal intensity of calcium signal in the hyphae near, middle and far from the cut site. mean ± SD; *n* = 5. C, D) Time variation of calcium signal propagation is indicated by different colors (C). Line profiles along arrow in (C) at different time are shown by different colors (D), mean ± SD; *n* = 3. E) Time course of signal intensity at different color boxes. Images of signal appearance at color boxes. The region is shown in (A) as the dot-line box. F) Images of calcium signal before and after cut the mycelial edge perpendicular to the hyphal growth direction from Video 2. The cut site is indicated by a gray line. G) Line profiles along the arrow. Mean ± SD; *n* = 3. H) Line profiles of calcium signal conduction in the 1-day, 2-, 7- and 14-days colonies. Mean ± SD; *n* = 3. Box plots of signals longest distance from the cut site. *n* = 14, 13 from 3, 4 independent experiments. I) The box area 800–1400 mm from the cut site. J) Image sequence of two frequent signal appearances. The region is shown in (I) as the dot-line box. K) Time course of signal intensity at different color circles in (J) at different time. L) Scatterplot of the time the signal appeared and the distance from the cut site. Different colors by different waves. M) Images of calcium signal after cut the mycelial edge in 7-days and 14-days colonies from Video 3. The cut site is indicated by a gray line. Line profiles along the arrow. Mean ± SD; *n* = 3. N) Image sequence of mycelial growth before and after the cut. O) Mycelial elongation rate at the area severed near hyphal tips, severed at the bottom of hyphae, and not severed. Mean ± SD; *n* = 3.

Intracellular Ca^2+^ content was visualized by red fluorescent Ca^2+^ biosensor, R-GECO, in the model fungus *A. nidulans* (16). The red fluorescence in the mycelium was monitored by a wide-field fluorescence microscope, when the colony, ∼10 mm diameter after 2 days incubation, was cut with a razor blade (13) (see Methods). We cut the mycelial edge was along the parallel direction to the hyphal growth to investigate how the signal spreads horizontally in mycelium to other adjacent hyphae (Fig. 1A, Video S1). Calcium signal appeared simultaneously at ∼500 μm from the cut site, peaked at 10 s (Figs. 1B and S1A, *t* = 33), and gradually faded until 120 s (Fig. 1C, D). The initial fluorescence was stronger near the cut (>300 μm), diminished as the distance increased from 300 to 600 μm, and showed no significant difference further than 600 μm (Fig. 1A–D). The signal was stable throughout the hyphae around the cleft (>300 μm). At 300–600 μm from the cut site, following the initial fluorescence’s faded, the signal often appeared, spread, and disappeared within the hyphae (Video S1). Initial fluorescence was low and quickly disappeared in mycelia far from the cut (<600 μm). The longest signal was detected at distance of ∼1,000 μm from the cut site (Fig. 1D).

Following the initial fluorescence’s faded, short-lived signals were observed at the limited area (<50 μm) of tips or in the middle of hyphae at various locations in the mycelium (Video S1). The Ca^2+^ signal appeared to propagate between different hyphae in close and spread within the mycelium (Figs. 1E and S1B). The signal intensity decreased as the distance from the cut site increased. There appears to be a correlation between signal appearance time and distance from the cleavage site (Fig. S1A). The same hyphae rarely blinked repeatedly.

When the similar size mycelium was cut around the edge vertically to the hyphal growth direction (Fig. 1F, G, Video S2), calcium signal immediately emerged throughout the mycelium near the cleavage site (<500 μm). The signal intensity decreased gradually as further away from 500 μm (Fig. S1C), which is like Fig. 1B. Around 1,000 μm from the cut site, a signal peak appeared in the middle of hyphae (Fig. 1G, H). This suggests that the diffuse signal from the cleavage extends to around 1,000 μm. The signal decreased drastically at further distance (Fig. S1D). In the box area 800–1,400 μm from the cut site, there were two frequent signal appearances, suggesting signal propagation by the first and second waves whose transfer rates were 41 and 50 μm/s, respectively (Fig. 1I–L).

The smaller colony, ∼5 mm diameter after 1 day incubation, was cut in half (Fig. S1E, F). Calcium signal immediately emerged throughout the mycelium near the cleavage site and weaker away from the cleavage. The signal appeared in the hyphae <700 μm from the cleavage. When the 2-day-cultured mycelia were cut at Fig. 1A and F, the signal diffused over 1,000 μm regardless of the cutting way, but when the younger mycelium was cut, the signal only traveled 700 μm (Fig. 1H). When 7- or 14-day-cultured mature mycelia were cut around the edge vertically, the signal diffused around 1,500 μm from the cleavage (Fig. 1H, M, Video S3). These results suggest that the distance of signal transmission depends on the maturity of the mycelial network and that calcium signals travel only 1,500 μm maximum in the mycelial network. The fluorescence intensity near the cleavage was comparable in 2-, 7-, and 14-day colonies and higher than that of 1 day colony (Fig. S1G). The analysis of effect by the cleavage on mycelial growth found mycelial elongation was delayed only in the area severed near hyphal tips, otherwise most were unaffected (Fig. 1N, O, Video S4).

### Conduction of calcium signaling in mycelium by drop of EtOH or NaCl

**Fig. 2.**
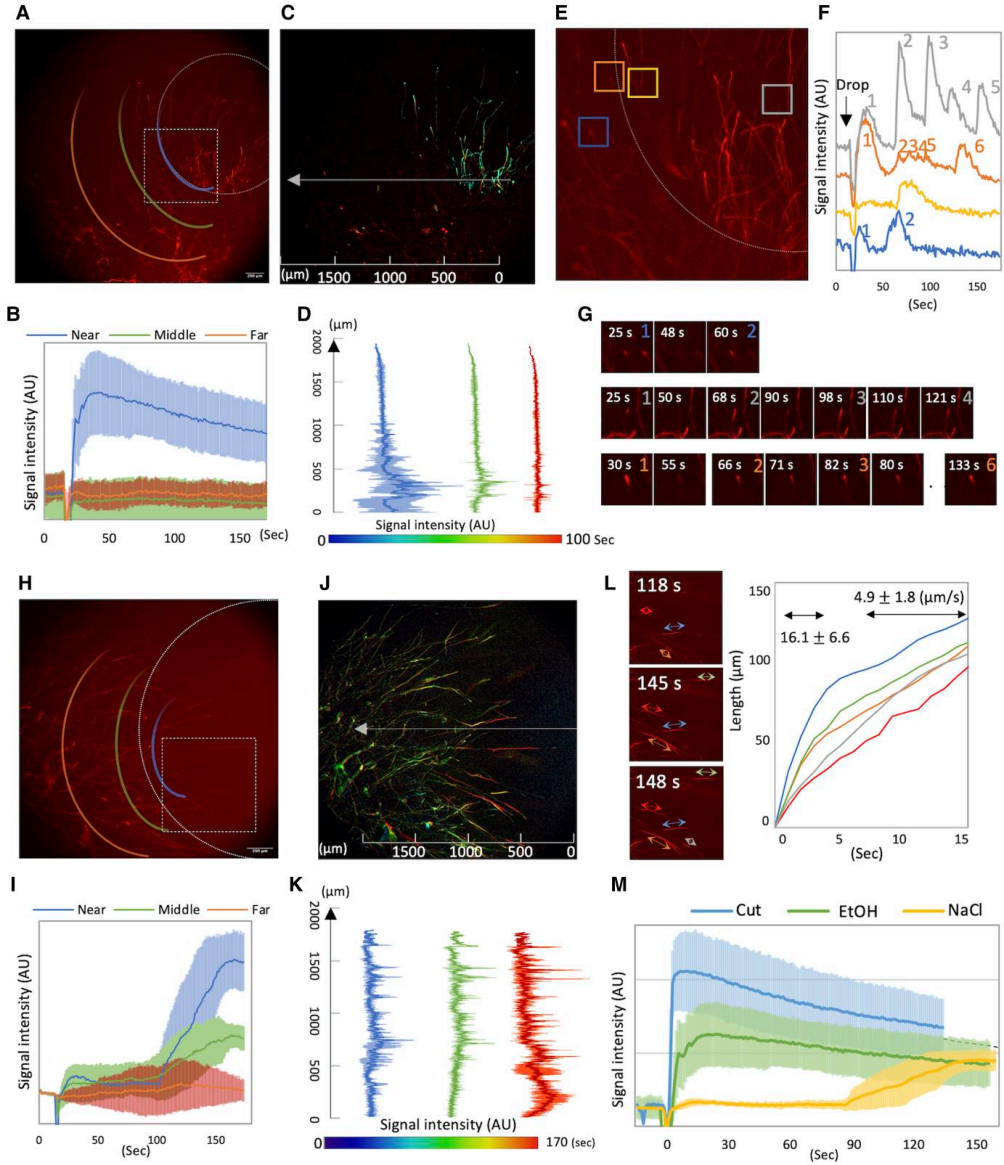
Conduction of calcium signal in the mycelia with a drop of EtOH or NaCl. A) Image of calcium signal immediately after a drop of EtOH, from Video S4. The drop site is indicated by a white circle. According to the distance from the drop site, the regions were classified as near, middle and far. B) Time course of signal intensity of calcium signal in the hyphae near, middle and far from the drop site. Mean ± SD; *n* = 5. C, D) Time variation of calcium signal propagation is indicated by different colors (C). Line profiles along arrow in (C) at different time are shown by different colors (D). Mean ± SD; *n* = 3. E) Blinking of calcium signal in the hyphae at color boxes. The region is shown in (A) as the dot-line box. F) Time course of signal intensity at different color boxes in (E). G) Image sequence and time course of signal intensity at the boxed in (E) are shown. Number of blinks are shown. H) Image of calcium signal immediately after a drop of NaCl. I) Time course of signal intensity of calcium signal in the hyphae near, middle and far from the drop site. Mean ± SD; *n* = 5. J, K) Time variation of calcium signal propagation is indicated by different colors (J). Line profiles along arrow in (J) at different time are shown by different colors (K). Mean ± SD; *n* = 3. L) Image sequence of spread of calcium signal in the hyphae. The spread is show by colored arrows. Time variation of diffusion distance are shown by colored line graph. M) Time course of signal intensity of calcium signal in the hyphae with cut, a drop of EtOH or NaCl. Mean ± SD; *n* = 5.

When 1 μL ethanol was dropped to the edge of mycelium as the dehydration stress, whose diameter is ∼10 mm, calcium signal appeared simultaneously in the dropped area, whose intensity reached its peak after 15 s, decreased slowly until 160 s (Fig. 2A, B, Video S5). The fluorescence hardly spread out the dropped area (Figs. 2C, D and S2A). The calcium signal blinked repeatedly at the tips of many hyphae. Some hypha blinked 5 times every 35 s for 175 s (Fig. 2E–G, gray), other blinked a quick weak signal 4 times in 30 s (orange *t* = 63–99). The maximum value of fluorescent intensity that blinked multiple times gradually decreased with time (Fig. 2F). Outside of the ethanol dropped area, hyphae rarely blinked, and the number of times they blinked was limited to two or three (Fig. S2B).

When 1 μL 3 M NaCl was dropped to the edge of mycelium as the salt stress, whose diameter is ∼10 mm, a few hyphal tips fluoresced in the dropped area and faded quickly (Fig. 2H, I, Video S6). After 80 s period of non-fluorescence, the fluorescent intensity of many hyphae gradually increased (Fig. 2H–K). The calcium signal spread from the middle of hyphae to both the tip and the base at a rate of 4.9 ± 1.8 μm/s (Figs. 2L and S2D). After 150 s, most of the hypha in the NaCl-dropped area showed stable fluorescence that was diffuse throughout the hyphae (Fig. 2J, K). The signal intensity gradually decreased until 300 s (Fig. S2G). Outside of the dropped area, a small number of hyphae briefly blinked one or two times (Fig. S2F). Similar signal patterns were observed in the 7-day-cultured colony in response to ethanol and NaCl (Fig. S2H, Video S7).

Comparing the time course of the stress responses of cleavage, ethanol, and NaCl, the cleavage reached its maximum value immediately (<5 s), while the ethanol stress reached its peak after a slight delay of 15 s. The cleavage showed a higher maximum than the ethanol or NaCl treatment (Fig. 2M). The NaCl stress showed an increase in signal after 90 s, reaching a peak comparable with the ethanol treatment. Mycelial elongation was delayed only in the area dropped EtOH or NaCl, otherwise most were unaffected (Fig. S2I, J, Video S3).

### Calcium signal conduction through hyphae

**Fig. 3.**
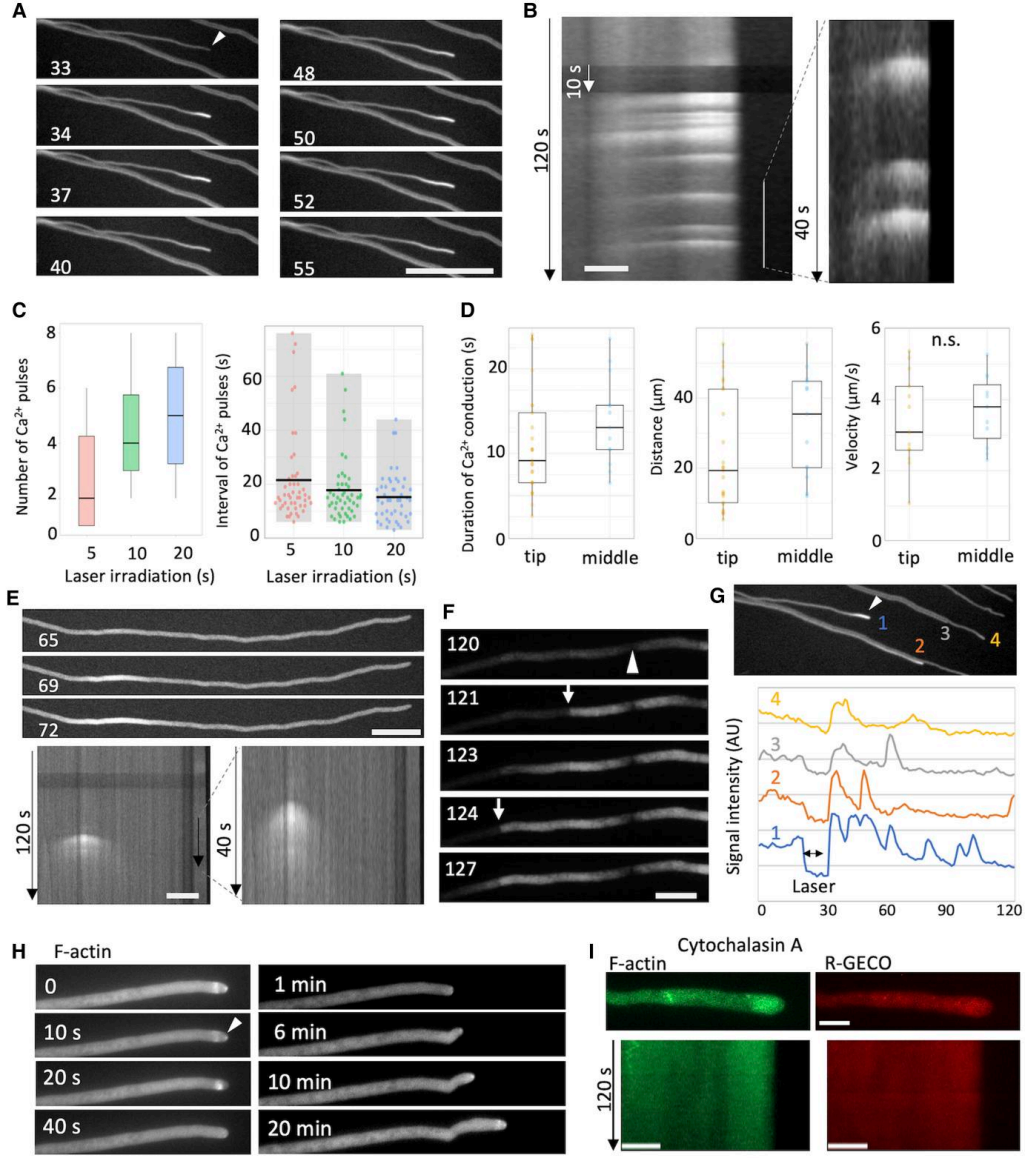
Calcium pulses in hyphae stimulated by point laser irradiation. A) Image sequence of calcium pulses after 10 s of pointed laser irradiation to the tip of the hypha (arrowhead) from Video S6. The elapsed time is given in seconds. Scale bar: 20 μm. (B) Kymograph along the hypha shown in (A). Total elapsed time 120 s. Scale bar: 20 μm. C) Box plots of the number of calcium pulse and the intervals of calcium pulses from hyphal tips after 5, 10, and 20 s laser irradiation (*n* = 10–14, *n* = 17–33, respectively). D) Box plots of duration, distance, and velocity of calcium singal from hyphal tips (*n* = 35 measurements for each) and at the middle of hyphae (*n* = 6 measurements for each). E) Image sequence of the calcium signal at the middle of the hypha from Video S7. The elapsed time is given in seconds. Scale bar: 20 μm. Total 120 s. F) Image sequence of calcium signal that reached and passed through the septa (arrows). The elapsed time is given in seconds. Scale bar: 20 μm. G) Ca^2+^ pulses in the surrounding hyphae (2–4) after the laser irradiation at (1, arrowhead). Line profiles of temporal changes in the calcium signal at each hyphal tip (1–4). Total 120 s. H) Image sequence of GFP-TpmA and change of growth direction after point laser irradiation (arrowhead). The elapsed time is given in seconds (left) and minutes (right). I) Images of F-actin and Ca^2+^ in hyphae treated with Cytochalasin A (actin polymerization inhibitor). Kymographs along the hyphae are shown. Total 120 s. Scale bar: 5 μm.

Calcium signal was analyzed in each hypha stimulated by point laser irradiation (see Methods). When the point laser was irradiated on the hyphal tip, calcium signals quickly appeared repeatedly, each signal spreading backward and disappearing (Fig. 3A, B, Video S8). Within 2 min, the signal appeared 4 ± 2 times (Fig. 3C). The kymograph analysis yielded the conduction time, distance, and velocity: 11 ± 5 s, 25 ± 15 μm, and 4 ± 2 μm/s (Fig. 3D), respectively. The mean interval time of calcium signals was 18 ± 12 s (*n* = 48 signal). As observed in Fig. 2L, calcium signal sometimes diffused in both directions at the middle of the hyphae, (Fig. 3E, Video S9). There was no significant change in conduction time and velocity between the tip and the middle of hyphae (Fig. 3D). The calcium signal pass through the septa, but it slowed once there (Fig. 3F, Video S9). Flickering calcium signals were transmitted to neighboring hyphae, but signal intensity and number of flickers decreased (Fig. 3G).

The number of calcium pulses and intervals were monitored when the laser irradiation time was changed to 5 and 20 s instead of 10 s. The average calcium pulses numbers were 3 ± 2, and 5 ± 2, respectively (Fig. 3C). The average interval times were 21 ± 17 and 15 ± 9 s, respectively (Fig. 3C). As laser irradiation time increased, the number of calcium pulses increased, and the interval became shorter. Without laser stimulation, the average interval between pulses was 26 ± 7 s (16).

The laser irradiation stopped the hyphal growth for a short time. A new polar site emerged from the tip after several minutes, and tip growth began. Laser irradiation caused F-actin and secretory vesicles at the apex of the mycelial tip to lose their signal (Figs. 3H and S3A, Video S10). When hyphal growth resumed, F-actin and secretory vesicles localized to the new polar site. The results suggest that calcium signal elicited by laser irradiation have a role in quick actin depolymerization and membrane transport pausing. In general, intracellular Ca^2+^ levels directly regulate actin assembly and vesicle fusion (18, 19). Conversely, when actin polymerization or membrane transport was inhibited by chemical treatment, hyphal growth ceased, and no periodic calcium influx was also observed (Figs. 3I and S3B, C).

### Target proteins of CaM and CaMKs

Although calcium signal plays a role in the rearrangement of the actin cytoskeleton and vesicle transport in hyphae, little is known about how calcium signal controls these processes. The *A. nidulans* genome possesses genes encoding sole calmodulin (*caM*) and three CaMKs (*cmkA*, *cmkB*, and *cmkC*) (Fig. S4A). *caM*, *cmkA*, and *cmkB* are essential since they are required for progression of mitosis (20–22). *cmkC* is not essential, but the spore germination and nuclear division are delayed, the colony is sensitive to osmotic stress (22, 23).

**Fig. 4.**
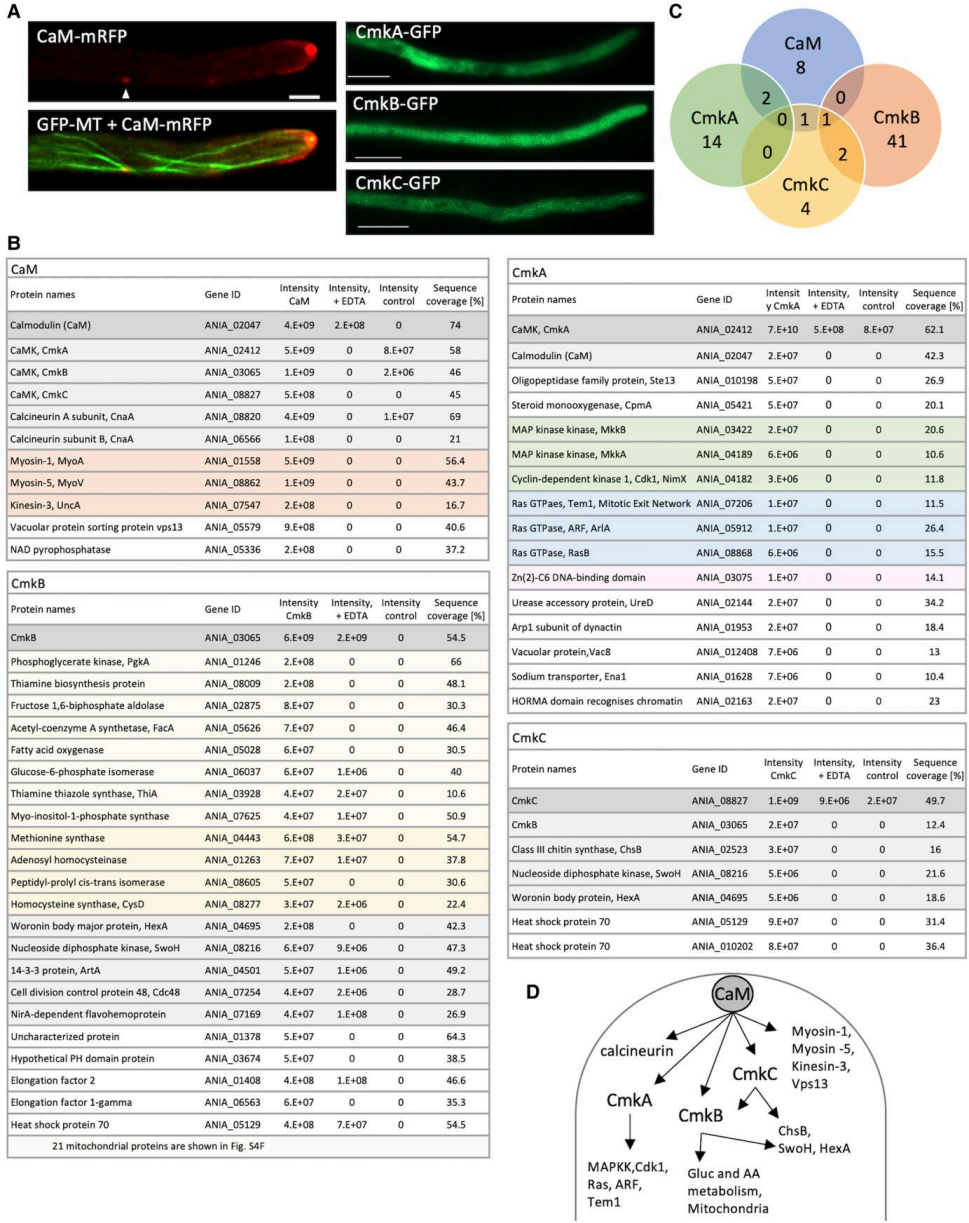
Localization and interacting proteins of CaM and CaMKs. A) Fluorescence image of GFP-labeled microtubules and CaM-RFP. Cytoplasmic localization of CmkA-GFP, CmkB-GFP, and CmkC-GFP in the hyphae. Scale bar: 10 μm. B) Proteins interacting with CaM, CmkA, CmkB, and CmkC. The table lists the protein name, gene ID, intensity of bait proteins and a negative control, and sequence coverage. The functional classification is shown by different colors. C) Venn diagram of proteins interacting with CaM, CmkA, CmkB, and CmkC. D) Summary of the calcium signaling pathway through CaM and CaMKs.

CaM-mRFP localized at the apex of growing hyphae and at the spindle pole body (Fig. 4A) (24, 25). Our movie shows CaM moved to the hyphal apex as microtubules elongated (Fig. S4B, Video S11). CmkA, CmkB, and CmkC, each with respective GFP tags and expressed under the native promoter, are present in the cytoplasm (Fig. 4A). These strains show no severe phenotype, indicating CaM-mRFP and CaMK-GFP fusion proteins are functional. qRT-PCR analysis did not detect any changes in the expression levels of CaMKs in response to osmotic stress and treatment with inhibitors of actin polymerization, membrane transport, or calcium channel (Fig. S4C). The apical localization of CaM-mRFP disappeared in response to EtOH or NaCl treatment (Fig. S4D).

CaM- and CaMK-interacting proteins were immunoprecipitated by using RFP-trap or GFP-trap and identified by LC-MS/MS (see Methods). SDS-PAGE confirmed that the bands of these proteins correspond to those of CaM and CaMKs (Fig. S4E). In two independent experiments, we focused on proteins having LC-MS/MS intensities that were 10-fold higher than the negative control (wild type), higher than 3.E + 06, and sequence coverages of >10% (Fig. 4B, Table S1). In the case of CmkB, proteins were focused with an intensity more than 4.E + 07 and sequence coverages >10% since many mitochondrial proteins were detected. CaM, CmkA, CmkB, and CmkC each have 11, 16, 44, and 7 potential target proteins, respectively. There was little overlap between target proteins (Fig. 4C), implying that signaling to distinct targets is functionally differentiated.

As CaM-mRFP-interacting proteins, we identified three CaMKs and calcineurin, a calmodulin-dependent phosphatase, subunits A and B (Fig. 4B). In addition, endocytosis and exocytosis motor proteins (myosin-1, myosin-5, and kinesin-3) (26–28) and an ortholog of the vacuolar sorting-associated protein Vps13 (29) were discovered. These proteins did not coprecipitate with CaM-mRFP when EDTA was added to the cell lysate, demonstrating that their interaction with CaM is Ca^2+^ dependent. GO and enrichment analysis (cluster frequency/background frequency) showed that proteins involved in vesicle trafficking and nuclear division regulation were enriched in the CaM-interacting proteins (Table S2).

CmkA-interacting proteins contain proteins involved in signal transduction, such as MAP kinase kinases (MkkA, MkkB) (23), Cyclin-dependent kinase (NimX, Cdk1) (30), and Ras GTPases, Tem1 for Mitotic Exit Network (31), ArlA (ARF; ADP ribosylation factor) for membrane traffic (32), and RasB for hyphal morphogenesis (33) (Fig. 4B). CmkB-interacting proteins are enriched in Acetyl-CoA biosynthesis, thiamine biosynthesis, gluconeogenesis, glycolytic process, and branched-chain amino acid metabolic process (Table S2), which are composed of both cytosolic and mitochondrial proteins. Probably because CmkB phosphorylates mitochondrial-associated proteins, mitochondrial fraction coprecipitated with CmkB in this experiment. Woronin body (HexA) (34), 14-3-3 ortholog ArtA (35), and a nucleoside diphosphate kinase (SwoH) (36) were also discovered, all of which play significant roles in hyphal development. CmkB co-precipitated with CmkC-GFP, that is consistent with that CmkC is CaMK kinase (CaMKK). ArtA and SwoH were identified again as CmkC-interacting proteins, although they could be indirect interaction via CmkB. Class III chitin synthase (ChsB), that is crucial for hyphal growth, co-precipitated with CmkC-GFP (37).

## Discussion

Intracellular Ca^2+^ dynamics have been shown in the hyphae of several filamentous fungi (16, 38, 39). This study is the first to show how calcium signals propagate within the mycelial network. Localized calcium signal was revealed when a piece of the mycelium was cut, and it spread outward for around 1,500 μm. Dropping ethanol or NaCl on a piece of the mycelium also activated the signal in the area where it was dropped. Mycelia lack a brain or nervous system, therefore the control is decentralized rather than centralized (2). Even if stress is perceived in one area of the mycelium, there is no need to alert the entire mycelium, rather, it is more efficient to respond to stress at that site. In fact, the growth of the colony was slowed only in the stressed areas, while most of the rest of the colony continued to grow without delay. The mycelium extending in all directions and making decisions at each location would be suitable for mycelial growth and adaptation.

Ca^2+^ oscillations are a ubiquitous means of signaling in all cells. Positive feedback, or in combination with negative feedback, can trigger the Ca^2+^ oscillations that arise from Ca^2+^-induced Ca^2+^ release (40). The shape of oscillations, which is characterized by their amplitude and phase, can efficiently transmit different cellular responses in both plant and mammalian cells (41). Our results indicate the shape of oscillations varies depending on the kinds of stress and proximity to the stress. As pointed laser irradiation time increased, the number of Ca^2+^ influxes increased, and the interval became shorter. The degree of the stress or the distance from the stress can activate differentially the downstream pathways, although deciphering that message requires further analysis.

In response to laser irradiation, actin cytoskeleton depolymerizes at the hyphal tip, which also stops vesicular transport and hyphal extension. After a short time, cell polarity and actin cytoskeleton are reconstituted, which resume vesicular transport and hyphal extension. Motor proteins (myosin-1, myosin-5, and kinesin-3) were identified as targets of CaM. The former is involved in actin organization, while the latter are involved in vesicle transport. CaM is localized at the hyphal tips, where actin polymerization and exocytosis occur, and at SPBs where the minus ends of the microtubule bind. CaM translocation to the hyphal tip is associated with microtubule elongation, which is expected to function in membrane transport in association with kinesin-3 and myosin-5. CmkA is also involved in membrane traffic through interaction with ArlA (ARF). ChsB, a class III chitin synthase, is transported by vesicle to hyphal tips and plays an essential role in hyphal tip growth (37, 42), which is co-precipitated with CmkC. Chitin synthase, Chs3, of *Candida albicans* is phosphorylated, and both phosphorylation and dephosphorylation are required for its correct localization and function (43). CmkC may be a link between calcium signaling and cell wall synthesis by phosphorylating the chitin synthase. Calcineurin is a target of the immunosuppressive drugs and is important in fungal virulence and drug resistance (44). Transcription factor Crz1, one of calcineurin targets, is involved in the regulation of stress response, cell wall integrity, and drug resistance through dephosphorylation and nuclear translocation (45). The cell wall integrity signaling pathway may also be involved in the stress response; however, since there is no overlap with that pathway other than ChsB (46), the functions are expected to be distinct from the calcium signaling pathway. Notably, a putative transcription factor is identified here as CmkA-associated proteins.

We have visualized the calcium oscillations in single hypha and have found a link to actin and exocytosis (16). We searched for targets of CaM and CamKs to reveal what molecules downstream of the calcium signal are involved in regulating hyphal growth. On the other hand, even if the response of a single hypha alone is clarified, since the response of overall mycelial network is unknown, we investigated how calcium signals are transmitted in the mycelial network. By exploring upstream and downstream of previous findings, respectively, this paper links phenomena at different hierarchies from macro mycelia to single hyphal cell, and to target proteins. Our results show that local calcium signal transmission in mycelial networks exhibits decentralized stress responses.

The mycelial network of Ascomycota *A. nidulans* was analyzed in this study. Given the differences in the fundamental structure between arbuscular mycorrhizal networks and Basidiomycota mycelial networks, we must be careful when determining if this finding applies to both. AM fungi optimize the mycelial networks by adjusting transport and distribution of nutrients during growth, so that flow is concentrated in the major hyphae, which become thicker and have thicker cell walls, whereas unnecessary hyphae become empty and are separated by septa (47). Ectomycorrhizal and saprotrophic basidiomycetes form mycelial strands, which are aggregates of multiple hyphae, and some form rhizomorphs with differentiated thick-walled fiber hyphae and vessel hyphae, with diameters of ∼10 to 15 μm, that are likely to improve flow (2, 48). It is still difficult to visualize the fluidity of mycelial networks, although there is growing evidence that quantum dots and fluorescent proteins can move within mycelial networks (49). Attempts are being made to predict the ecological importance of mycelial networks by modeling their development and features (50). Optimization of logistics in mycelial networks appears to be related to fungal memory (51). Electrical signaling in mycelial networks may also be involved in intelligent communication in fungi (52). There is a wide and unexplored field of research behind the study of the transmission of materials and information in mycelial networks, which reveals fundamental properties of fungi.

## Materials and methods

### Fungal strains and media

The strains of filamentous fungi used in this study are listed in Table S3. Tagging with GFP is described in Supplemental Methods and Table S4.

### Ca^2+^ imaging in mycelia

*Aspergillus nidulans* expressing the genetically-encoded Ca^2+^ biosensor R-GECO was imaged with a motorized fluorescence stereo microscope (SMZ-25; Nikon) equipped with P2-SHR PLAN APO 1x/0.16 objective lens (Nikon) and an sCMOS camera (ORCA-Flash4.0 V2; Hamamatsu Photonics) (13, 53). R-GECO was excited using a mercury lamp (Intensilight Hg Illuminator; Nikon), a 561/14-nm excitation filter (FF01-561/14-25, Semrock), and a 575-nm dichroic mirror (Di02-R561-25 × 36; Semrock). The red fluorescent signal passing through a 609/54-nm filter (FF01-609/54-25; Semrock) was acquired with the sCMOS camera using NIS-Elements imaging software (Nikon).

### Point laser irradiation

*Aspergillus nidulans* cells were grown on minimal medium agar plates at 30°C for 2 days. Then, the edges of colonies were cut into 5 mm squares and placed in glass dishes for observation under an inverted microscope IX-83 (Olympus). A localized heat shock was imposed on individual hypha using an IR-LEGO 1000 system (Sigma Koki) (54, 55), equipped with custom-made UPlanSApo 20x/0.75 and UPlanSApo 40x/0.95 objective lens (Olympus), operating at 15 mW for 5–20 s. MetaMorph and Image J software were used for image analysis.

### GFP-trap and protein identification by Lc**-**MS**/**Ms

All information is shown in Supplemental information. The proteomic data (Table S1) have been deposited in the ProteomeXchange Consortium via the jPOSTrepo with the data set identifier PXD027777 for ProteomeXchange and JPST001285 for jPOSTrepo.

Other methods are shown in supplemental information.

## Supplementary Material

pgad012_Supplementary_Data

## Data Availability

All data that support the findings of this study are available in this manuscript and the Supplementary Materials. Raw data for proteomic analysis have been deposited in the ProteomeXchange Consortium via the jPOSTrepo with the data set identifier PXD027777 for ProteomeXchange and JPST001285 for jPOSTrepo.
