# Supplementary material for: Local calcium signal transmission in mycelial network exhibits decentralized stress responses
Source: PNAS Nexus. 2023 Mar 7;2(3):pgad012. doi: 10.1093/pnasnexus/pgad012 (PMC9991499; doi:10.1093/pnasnexus/pgad012)
Supplement: pgad012_Supplementary_Data [file pgad012_supplementary_data.zip › PNASNEXUS-PNASNEXUS-2022-00815-s01.pdf]

Supplemental information: Supplemental Methods, Supplemental Figure 1-4, Supplemental Table 1-4, Video legends

Supplemental Methods

Fungal strains and media. The strains of filamentous fungi used in this study are listed in Table S3. The composition of the supplemented minimal medium for *A. nidulans* and the procedures for standard strain construction have been described previously (1). For the overexpression of R-GECO, we used 2% threonine as the carbon source. The media were supplemented with 1 μM $CaCl_2$ to facilitate the detection of R-GECO signals.

Tagging with GFP. To tag CmkA with GFP at the C terminus, we amplified the 1-kb 5′ -UTR of *cmkA* and the 1-kb 3′ -UTR *cmkA* using genomic DNA of *A. nidulans* as the template and primer sets 7 and 4 and sets 5 and 8, respectively (primers used in this study are listed in Table S4). DNA fragments encoding GFP and Af-*pyrG* were amplified using GA5-GFP-Af-pyrG plasmid as a template and primer sets 1 and 2. The resulting three DNA fragments were combined by fusion-PCR using primer sets 3 and 2. Similarly, we used primer sets 9-20 to tag CmkB and CmkC with GFP at the C terminus. The PCR cassettes were used to transform the wild-type strain, TN02A3, and the transformants were confirmed by PCR (Fig. S4G).

RNA isolation, cDNA preparation, and quantitative RT-PCR. Strain TN02A3 was cultured in minimal medium for 24 h. Then, the mycelia were treated with 4.2 μM (2 μg/ml) Cytochalasin A, 3.5 μM (1 μg/ml) Brefeldin A, or 5 μg/ml Ionophore A23187 for 2 h. Another set of mycelia were shifted to minimal medium containing 0.6 M KCl for 2 h. All treated mycelia were frozen in liquid nitrogen, and then total RNA was isolated using an RNeasy Plant mini kit (QIAGEN). The cDNA pools from total RNA were generated by reverse transcription using a Prime Script RT Master Mix (TAKARA). RT-PCR was performed using a THUNDERBIRD SYBR qPCR Mix kit (Toyobo). Primer sets 21–30 (Table S4) were used for quantitative PCR. The actin gene *actA* was used as a normalization reference (internal control) for target gene expression level analysis. Each sample was analyzed in triplicate.

Point laser irradiation. *A. nidulans* cells were grown on minimal medium agar plates at 30 °C for 2 days. Then, the edges of colonies were cut into 5 mm squares and placed in glass dishes for observation under an inverted microscope IX-83 (Olympus). A localized heat shock was imposed on individual hypha using an IR-LEGO 1000 system (Sigma Koki)(2, 3), equipped with custom-made UPlanSApo 20x/0.75 and UPlanSApo 40x/0.95 objective lens (Olympus), operating at 15 mW for 5–20 s. MetaMorph and Image J software were used for image analysis.

Microscopy. Cells were grown in eight-well glass-bottom slides (Ibidi) with 500 μl minimal medium at 30 °C overnight. The mycelia were treated with either 4.2 μM (2 μg/ml) Cytochalasin A or 3.5 μM (1 μg/ml) Brefeldin A for 30 min, or shifted to a minimal medium with 0.6 M KCl for 30 min. The mycelia were observed using an inverted epi-fluorescence microscope (Axio Observer Z1, Carl Zeiss) equipped with a Plan-Apochromat 63 × 1.4 oil objective lens, an AxioCam 506 monochrome camera, and Colibri.2 LED (Carl Zeiss) as a light source. The temperature of the stage was kept at 30 °C by a thermo-plate (TOKAI HIT, Japan). Images were collected and analyzed by using the Zen system (Carl Zeiss) and ImageJ software.
Confocal images were acquired using a confocal laser scanning microscope (CLSM) LSM880 (Carl Zeiss, Jena, Germany) equipped with an Airyscan system for higher resolution, a 63×/0.9 numerical aperture Plan-Apochromat objective, and a 40x/0.75 numerical aperture IR Achroplan W water immersion objective (Carl Zeiss). Confocal images were acquired from CaM-RFP and GFP-labeled microtubules irradiated with 488- and 633-nm lasers, respectively. The acquired confocal images were analyzed using ZEN Software (Version 3.5, Carl Zeiss) and ImageJ software.

GFP-trap. Strains CSA02, STS02, STS03, and STS04 were cultured in minimal medium for 24 h, and then the mycelia were frozen with liquid nitrogen and ground using a mortar and pestle. The resulting powdered mycelia were placed in 10 mL of B+-buffer (300 mM NaCl, 100 mM Tris at pH 7.5, 10 % glycerol, 1 mM EDTA, 0.1 % NP-40, with protease inhibitor and DTT added immediately before use) and mixed with PMSF. The mixture was centrifuged for 10 min, and then the supernatant was equilibrated with GFP-Trap Agarose (Cosmo Bio) and then mixed by inversion at 4 °C for 1–2 h. The mixture was centrifuged at 1,500 rpm for 1 min, and then the beads were washed twice with 5 mL of B+-buffer. All beads were then transferred to new microcentrifuge tubes using 1 mL of B+-buffer. The beads were washed twice with 1 mL B+-buffer. The beads were then suspended in 50 mL of 2× SDS-sample buffer, and the mixture was boiled at 95 °C for 10 min and then centrifuged at 2500 G for 2 min. The supernatant was analyzed by SDS-PAGE.

Protein identification by LC-MS/MS. Former samples (each 15 μl) were loaded on SDS-PAGE gels (10% acrylamide) with stacking gels. Samples within the separating gel were excised and digested with trypsin. Peptide solutions were desalted using SPE-C18 tips (NTCR-KT200-C18, Nikkyo Technos, Tokyo, Japan) and then lyophilized. The lyophilized peptides were dissolved with 0.1% TFA in 90% acetonitrile and then separated and analyzed using a nano LC system (nano-Advance HPLC, Bruker) equipped with a Zaplous alpha Pep C18 column (0.1 x 150 mm, 3 μm, 120 Å, AMR Inc.) and a nano-ESI-Orbitrap MS (Q-Exactive Plus, Thermo Scientific). The initial mobile phase was 5% solvent A (0.1 % formic acid and 2% acetonitrile), with the concentration of solvent B (0.1% formic acid and 98% acetonitrile) increasing to 50% for 25 min.

The concentration of solvent B was increased to 95% for 1 min and then maintained at that concentration for another 4 min. The column was re-equilibrated for 5 min. The ion source polarity was set in the positive ion mode with a resolution of 70,000. MS/MS spectra were obtained with the data-dependent acquisition mode set at Top15. LC-MS/MS analysis was repeated three times for each sample. Proteins were identified and quantified using MaxQuant ver 1.6.0.16 (2). Reference sequence data were obtained from the Swis-Prot and TrEMBL databases. Peptide intensities obtained from each sample were normalized using the iBAQ algorithm (3). The proteomic data (Table S1) have been deposited in the ProteomeXchange Consortium via the jPOSTrepo (4) with the data set identifier PXD027777 for ProteomeXchange and JPST001285 for jPOSTrepo.

References

1. Takeshita N, Mania D, Herrero S, Ishitsuka Y, Nienhaus GU, Podolski M, Howard J, Fischer R. 2013. The cell-end marker TeaA and the microtubule polymerase AlpA contribute to microtubule guidance at the hyphal tip cortex of *Aspergillus nidulans* to provide polarity maintenance. *J Cell Sci.* 126:5400-11.
2. Tyanova S, Temu T, Cox J. 2016. The MaxQuant computational platform for mass spectrometry-based shotgun proteomics. *Nat Protoc.* 11:2301-2319.
3. Schwanhäusser B, Busse D, Li N, Dittmar G, Schuchhardt J, Wolf J, Chen W, Selbach M. 2011. Global quantification of mammalian gene expression control. *Nature*. 473:337-42.
4. Okuda S, Watanabe Y, Moriya Y, Kawano S, Yamamoto T, Matsumoto M, Takami T, Kobayashi D, Araki N, Yoshizawa AC, Tabata T, Sugiyama N, Goto S, Ishihama Y. 2017. jPOSTrepo: an international standard data repository for proteomes. *Nucleic Acids Research.* 45:D1107-D1111.

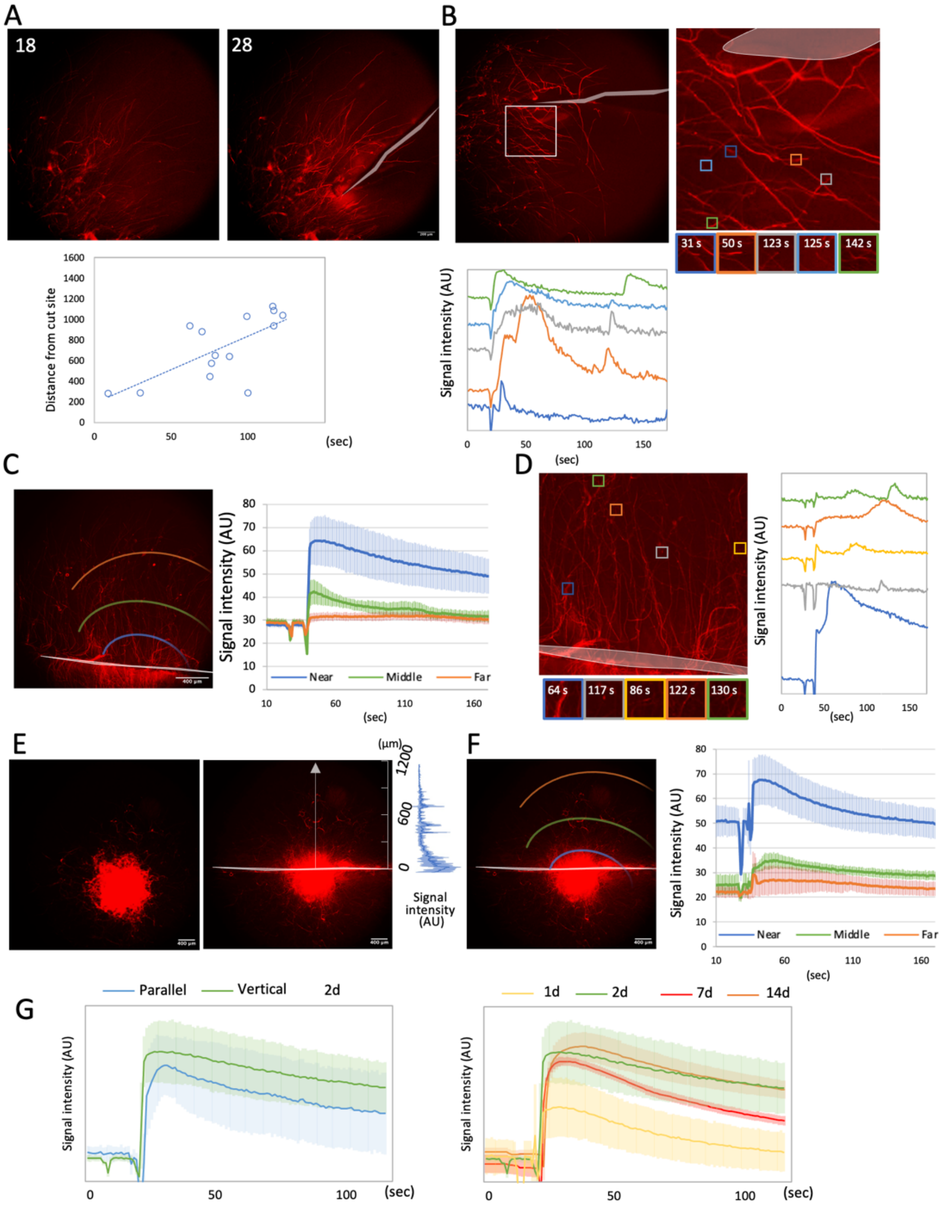

Figure S1. Conduction of calcium signal in the cut mycelia. (A) Images of calcium signal before and after cutting the mycelium, from Video 1. The elapsed time is given in seconds. Scatterplot of the time the signal appeared and the distance from the cut site. (B) Time course of signal intensity at different color boxes. Images of signal appearance at color boxes. The region is shown in left image as a line box. (C) The cut site is indicated by a white area. According to the distance from the cut site, the regions were classified as near (blue), middle (green) and far (orange). Time course of signal intensity of calcium signal in the hyphae near, middle and far from the cut site. mean ± SD; n = 5. (D) Time course of signal intensity at different color boxes. Images of signal appearance at color boxes. The region is shown in left image as a line box. (E) Image of calcium signal immediately after cutting half in the smaller colony. The cut site is indicated by a white area. Line profiles along the arrow. (F) Time course of signal intensity of calcium signal in the hyphae near, middle and far from the cut site. mean ± SD; n = 5. (G) Time course of signal intensity of calcium signal in the 1-day, 2-, 7- and 14-days colonies. mean ± SD; n = 5.

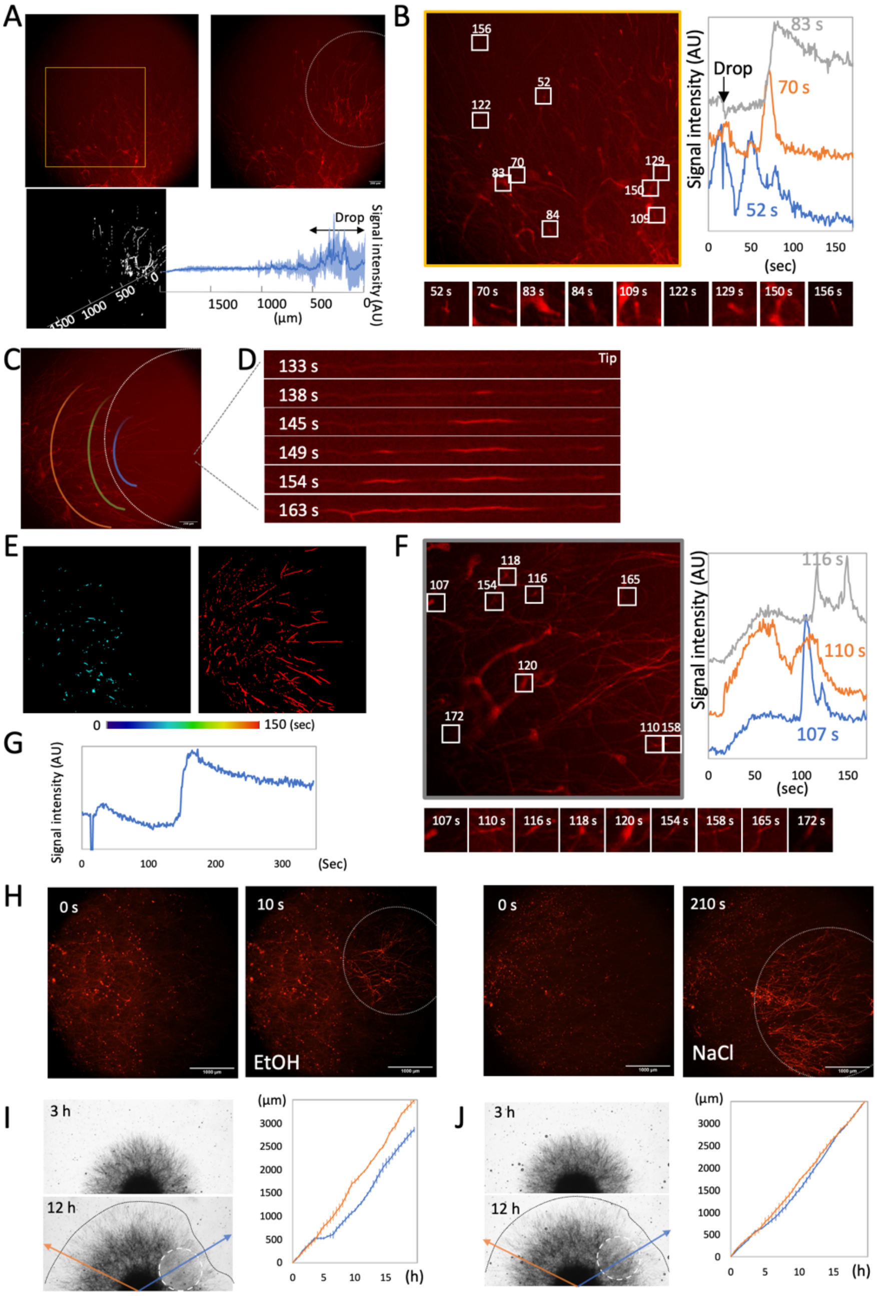

Figure S2. Conduction of calcium signal in the mycelia with a drop of EtOH or NaCl. (A) Images of calcium signal before and after a drop of EtOH. Line profiles along an arrow in the difference image between two. mean ± SD; n = 3. (B) Time course of signal intensity at different boxes. Images of signal appearance at boxes. The elapsed time is given in seconds. The region is shown in (A) as a line box. (C) Image of calcium signal immediately after a drop of NaCl, from Video 5. (D) Image sequence of spread of calcium signal in the hyphae. (E) Time variation of calcium signal propagation is indicated by different colors. (F) Time course of signal intensity at different boxes. Images of signal appearance at boxes. The elapsed time is given in seconds. The region is shown in (C) as a line box. (G) Time course of signal intensity of calcium signal in the hyphae near the drop site. (H) Images of calcium signal before and after a drop of EtOH or NaCl in the 7-days cultured colonies. (I, J) Mycelial elongation rate near a drop of EtOH (I) or NaCl (J) and except that are indicated by blue and orange lines. mean ± SD; n = 3.

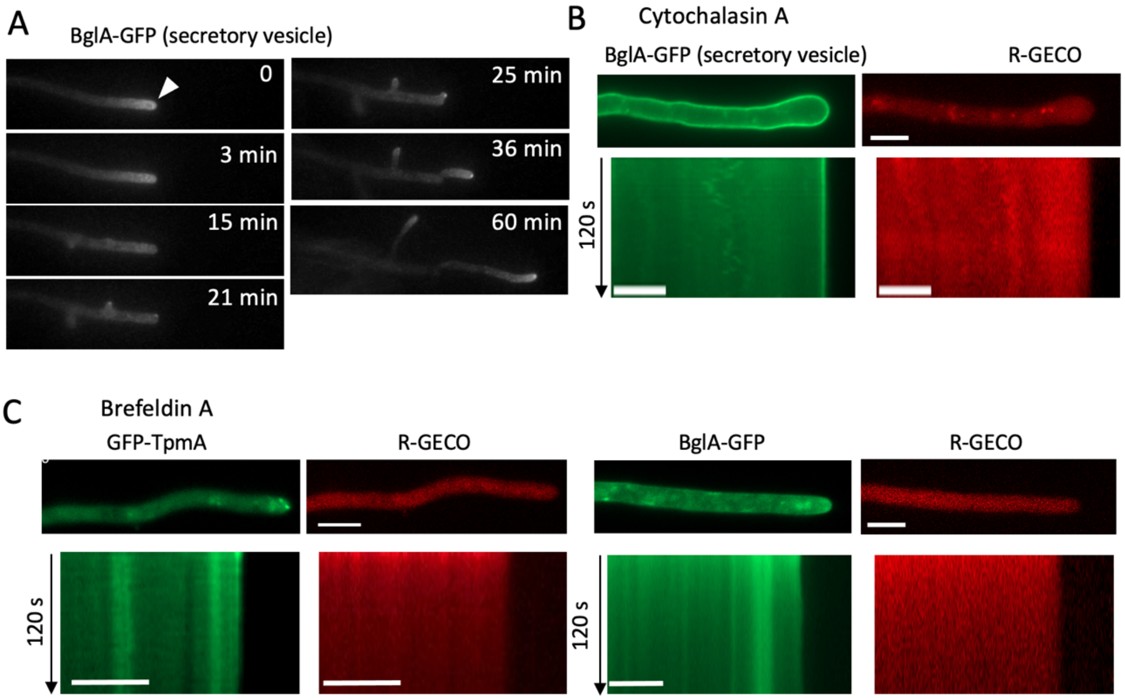

Figure S3. Interdependence between actin polymerization, exocytosis, and Ca^{2+} pulses. (A) Image sequence of BglA-GFP and change of growth direction after point laser irradiation (arrowhead). The elapsed time is given in minutes. (B, C) Fluorescence images of F-actin, secretory vesicles, and Ca^{2+} visualized by GFP-TpmA, BglA-GFP, and R-GECO, respectively, in hyphae treated with Cytochalasin A (actin polymerization inhibitor, 2 μg/ml, 30 min) (B), Brefeldin A (ER-Golgi membrane traffic inhibitor, 1 μg/ml, 30 min) (C). Scale bar: 5 μm. Kymographs along the hyphae are shown. Total 120 s. Scale bar: 5 μm.

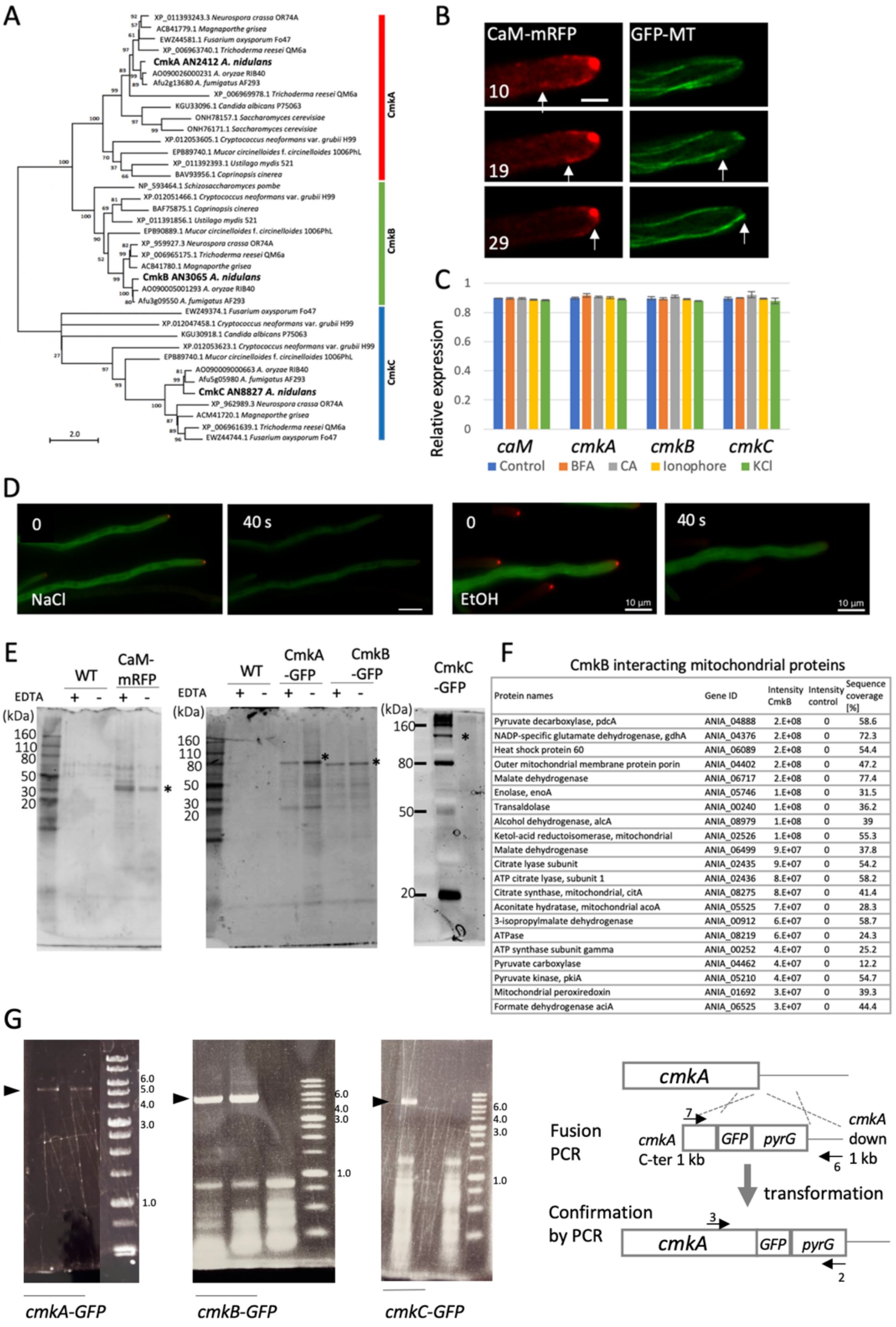

Protein names	Gene ID	Intensity CmkB	Intensity control	Sequence coverage [%]
Pyruvate decarboxylase, pdcA	ANIA_04888	2.E+08	0	58.6
NADP-specific glutamate dehydrogenase, gdhA	ANIA_04376	2.E+08	0	72.3
Heat shock protein 60	ANIA_06089	2.E+08	0	54.4
Outer mitochondrial membrane protein porin	ANIA_04402	2.E+08	0	47.2
Malate dehydrogenase	ANIA_06717	2.E+08	0	77.4
Enolase, enoA	ANIA_05746	1.E+08	0	31.5
Transaldolase	ANIA_00240	1.E+08	0	36.2
Alcohol dehydrogenase, alcA	ANIA_08979	1.E+08	0	39
Ketol-acid reductoisomerase, mitochondrial	ANIA_02526	1.E+08	0	55.3
Malate dehydrogenase	ANIA_06499	9.E+07	0	37.8
Citrate lyase subunit	ANIA_02435	9.E+07	0	54.2
ATP citrate lyase, subunit 1	ANIA_02436	8.E+07	0	58.2
Citrate synthase, mitochondrial, citA	ANIA_08275	8.E+07	0	41.4
Aconitate hydratase, mitochondrial acoA	ANIA_05525	7.E+07	0	28.3
3-isopropylmalate dehydrogenase	ANIA_00912	6.E+07	0	58.7
ATPase	ANIA_08219	6.E+07	0	24.3
ATP synthase subunit gamma	ANIA_00252	4.E+07	0	25.2
Pyruvate carboxylase	ANIA_04462	4.E+07	0	12.2
Pyruvate kinase, pkiA	ANIA_05210	4.E+07	0	54.7
Mitochondrial peroxiredoxin	ANIA_01692	3.E+07	0	39.3
Formate dehydrogenase aciA	ANIA_06525	3.E+07	0	44.4

Figure S4. Analyses of CaM and CaMKs. (A) Phylogenetic tree of CaMKs in several fungi. Amino acid sequences that showed >30% identities to CmkA, CmkB and CmkC, respectively, were obtained from the genome databases of respective organisms. Maximum likehood with 1000 bootstrap replicates was used for the tree construction. (B) Fluorescence image sequence of GFP-labeled microtubules and CaM-RFP. The elapsed time is given in seconds. (C) Expression levels of *caM, cmkA, cmkB*, and *cmkC* measured by qRT-PCR in the control (blue), and in treatments with Brefeldin A (1 μg/ml for 30 min) (orange), cytochalasin A (2 μg/ml for 30 min) (gray), the ionophore A23187 (10 μM for 15 min) (yellow), and KCl (0.6 M for 30 min) (green). Error bar: S.D., n = 3. (D) Images of GFP-labeled microtubules and CaM-RFP before and after a drop of EtOH or NaCl. (E) SDS-PAGE of CaM-RFP, CmkA-GFP, CmkB-GFP, and CmkC-GFP and their interacting proteins purified by RFP- or GFP-trap. (F) List of CmkB interacting mitochondrial proteins. (G) The scheme of GFP tagging at *cmkA* C-terminus. The fusion PCR, transformation and confirmation by PCR are indicated. The genetic integration of the PCR products into the targeted loci was confirmed by PCR.

Supplemental Table 1-4.

Table S1. The proteomic data have been deposited in the ProteomeXchange Consortium via the jPOSTrepo with the data set identifier PXD027777 for ProteomeXchange and JPST001285 for jPOSTrepo.

Table S2. GO and enrichment analysis.

	Biological process	Term	Count	%	P-Value	Fold Enrichment	Benjamini
CaM	GOTERM_BP_DIRECT	vesicle transport along actin filament	2	20	2.30E-03	789.7	6.40E-02
	GOTERM_BP_DIRECT	regulation of nuclear division	2	20	3.40E-03	526.4	6.40E-02
	GOTERM_BP_DIRECT	actin filament organization	2	20	2.80E-02	63.2	3.50E-01
	GOTERM_BP_DIRECT	protein phosphorylation	2	20	5.70E-02	30.4	5.40E-01
	GOTERM_BP_DIRECT	cellular response to oxidative stress	2	20	9.30E-02	18.4	7.10E-01
CmkA	GOTERM_BP_DIRECT	activation of MAPK activity	2	14.3	4.20E-03	430.7	1.40E-01
	GOTERM_BP_DIRECT	protein phosphorylation	2	14.3	7.10E-02	24.8	1.00E+00
	GOTERM_BP_DIRECT	cell division	2	14.3	9.20E-02	19	1.00E+00
CmkB	GOTERM_BP_DIRECT	tricarboxylic acid cycle	6	11.3	4.10E-06	23.7	4.70E-04
	GOTERM_BP_DIRECT	cellular response to farnesol	6	11.3	1.00E-05	19.8	4.70E-04
	GOTERM_BP_DIRECT	glycolytic process	5	9.4	1.10E-05	33.8	4.70E-04
	GOTERM_BP_DIRECT	gluconeogenesis	4	7.5	1.10E-04	40.6	3.50E-03
	GOTERM_BP_DIRECT	translation	7	13.2	5.50E-04	6.6	1.40E-02
	GOTERM_BP_DIRECT	carbon utilization	3	5.7	5.90E-03	25.1	1.30E-01
	GOTERM_BP_DIRECT	mitochondrial genome maintenance	3	5.7	1.30E-02	17.1	2.30E-01
	GOTERM_BP_DIRECT	cellular response to osmotic stress	3	5.7	1.80E-02	14.2	2.90E-01
	GOTERM_BP_DIRECT	acetyl-CoA biosynthetic process	2	3.8	2.10E-02	94.8	3.00E-01
	GOTERM_BP_DIRECT	thiamine biosynthetic process	2	3.8	4.10E-02	47.4	5.30E-01
	GOTERM_BP_DIRECT	anisotropic cell growth	2	3.8	4.70E-02	40.6	5.60E-01
	GOTERM_BP_DIRECT	branched-chain amino acid metabolic process	2	3.8	7.30E-02	25.8	7.90E-01
	GOTERM_BP_DIRECT	nucleosome assembly	2	3.8	8.00E-02	23.7	8.00E-01
	GOTERM_BP_DIRECT	maturation of LSU-rRNA	2	3.8	9.20E-02	20.3	8.60E-01
CmkC	GOTERM_BP_DIRECT	regulation of nuclear division	2	50	1.30E-03	1184.5	1.60E-02

	Cellular components	Term	Count	%	P-Value	Fold Enrichment	Benjamini
CaM	GOTERM_CC_DIRECT	vesicle	2	20	2.90E-03	612.9	7.00E-02
	GOTERM_CC_DIRECT	myosin complex	2	20	4.80E-03	367.8	7.00E-02
	GOTERM_CC_DIRECT	actin cytoskeleton	2	20	5.70E-03	306.5	7.00E-02
	GOTERM_CC_DIRECT	cytoplasm	5	50	1.20E-02	4.2	1.10E-01
	GOTERM_CC_DIRECT	hyphal tip	2	20	3.10E-02	55.7	2.30E-01
CmkA	GOTERM_CC_DIRECT	hyphal tip	3	21.4	8.60E-04	60.8	1.50E-02
	GOTERM_CC_DIRECT	spindle pole body	2	14.3	3.50E-02	51.4	3.00E-01
CmkB	GOTERM_CC_DIRECT	extracellular region	14	26.4	2.90E-07	5.8	5.30E-06
	GOTERM_CC_DIRECT	cytosol	15	28.3	3.00E-07	5.2	5.30E-06
	GOTERM_CC_DIRECT	cytosolic large ribosomal subunit	6	11.3	2.50E-05	16.5	2.90E-04
	GOTERM_CC_DIRECT	nucleosome	3	5.7	1.30E-03	53.3	1.10E-02
	GOTERM_CC_DIRECT	mitochondrial nucleoid	3	5.7	2.30E-03	40	1.60E-02
	GOTERM_CC_DIRECT	cytosolic small ribosomal subunit	3	5.7	1.80E-02	14.1	1.10E-01
	GOTERM_CC_DIRECT	mitochondrion	8	15.1	2.90E-02	2.6	1.50E-01
	GOTERM_CC_DIRECT	mitochondrial matrix	3	5.7	7.10E-02	6.7	3.10E-01

	Molecular Function	Term	Count	%	P-Value	Fold Enrichment	Benjamini
CaM	GOTERM_MF_DIRECT	calmodulin binding	4	40	1.30E-08	598.7	2.30E-07
	GOTERM_MF_DIRECT	calmodulin-dependent protein kinase activity	3	30	2.60E-06	898	2.30E-05
	GOTERM_MF_DIRECT	ATP binding	6	60	3.10E-04	7.1	1.90E-03
	GOTERM_MF_DIRECT	actin-dependent ATPase activity	2	20	2.00E-03	898	7.10E-03
	GOTERM_MF_DIRECT	microfilament motor activity	2	20	2.00E-03	898	7.10E-03
	GOTERM_MF_DIRECT	actin filament binding	2	20	3.40E-02	51.3	1.00E-01
	GOTERM_MF_DIRECT	calcium ion binding	2	20	6.40E-02	27.2	1.60E-01
	GOTERM_MF_DIRECT	protein serine/threonine kinase activity	2	20	7.60E-02	22.7	1.70E-01
CmkA	GOTERM_MF_DIRECT	MAP kinase kinase activity	2	14.3	4.10E-03	449	9.00E-02
	GOTERM_MF_DIRECT	GTPase activity	3	21.4	9.50E-03	18.2	9.50E-02
	GOTERM_MF_DIRECT	ATP binding	5	35.7	1.50E-02	4.4	9.50E-02
	GOTERM_MF_DIRECT	GTP binding	3	21.4	1.70E-02	13.3	9.50E-02
	GOTERM_MF_DIRECT	protein kinase activity	2	14.3	9.40E-02	18.7	4.10E-01
CmkB	GOTERM_MF_DIRECT	structural constituent of ribosome	8	15.1	9.10E-05	7.2	8.20E-03
	GOTERM_MF_DIRECT	magnesium ion binding	4	7.5	9.30E-03	9	3.60E-01
	GOTERM_MF_DIRECT	ATP citrate synthase activity	2	3.8	1.20E-02	161.6	3.60E-01
	GOTERM_MF_DIRECT	protein heterodimerization activity	3	5.7	2.20E-02	12.8	5.00E-01
	GOTERM_MF_DIRECT	L-malate dehydrogenase activity	2	3.8	3.00E-02	64.7	5.40E-01
	GOTERM_MF_DIRECT	ATP binding	10	18.9	3.60E-02	2.1	5.40E-01
	GOTERM_MF_DIRECT	metal ion binding	7	13.2	9.10E-02	2.2	1.00E+00
CmkC	GOTERM_MF_DIRECT	calmodulin-dependent protein kinase activity	2	50	1.10E-03	1347	7.80E-03
	GOTERM_MF_DIRECT	ATP binding	3	75	2.50E-02	8	6.80E-02
	GOTERM_MF_DIRECT	protein serine/threonine kinase activity	2	50	2.90E-02	51.2	6.80E-02

Table S3. *A. nidulans* strains used in this study.

Strain	Genotype
TN02A3	*pyrG89; argB2; Δ nkuA::argB; pyroA4*
SNT163	*pyrG89; argB2; Δ nkuA::argB; pyroA4; [pyroA] [alcA(p)-gfp-tpmA::pyr-4] ; [alcA(p)-r-geco::pyr-4]*
SNT164	*pyrG89; argB2; Δ nkuA::argB; pyroA4; [pyroA] [bglA-gfp::pyr-4] ; [alcA(p)-r-geco::pyr-4]*
CSA02	*pyrG89; pyroA4, Δ nkuA::argB2; cam::mRFP1::pyrG;riboB2*
STS1	*pyrG89; argB2; Δ nkuA::argB; cmkA::GFP::pyrG*
STS2	*pyrG89; argB2; Δ nkuA::argB; cmkB::GFP::pyrG*
STS3	*pyrG89; argB2; Δ nkuA::argB; cmkC::GFP::pyrG*
STS4	*Δ nkuA::argB2; cam::mRFP1::pyrG; alcA(p)-gfp-tubA::pyr-4*

Table S4. Primers used in this study

Primer	No.	Sequence（5’-3’）
ga5 fw	1	GGAGCTGGTGCAGGCGCTG
pyrG rv	2	CTGTCTGAGAGGAGGCACTG
cmkA up fw	3	CTGCAACTGAGCGCCTGC
cmkA up rv	4	CCAGCGCCTGCACCAGCTCCTGAGTGAGCCCGCTCGCGAG
cmkA dw fw	5	CAGTGCCTCCTCTCAGACAGAACCTCATATTGGGCGGTTG
cmkA dw rv	6	CTTTCGGCAGGGCCGCG
cmkA up fw nest	7	TCGTCAATGCTAGAGTGTGG
cmkA dw rv nest	8	CGAAGATCTGCGGCATGG
cmkB up fw	9	CCTTCCTGTAGATTTACCGC
cmkB up rv	10	CCAGCGCCTGCACCAGCTCCCCGTTCACTCCGCGGCGCAG
cmkB dw fw	11	CAGTGCCTCCTCTCAGACAGTGACTTGAGCTGCACCTGAT
cmkB dw rv	12	GGACTTGAGAAGCCATCTC
cmkB up fw nest	13	CGTACTACCGTCGAGGAA
cmkB dw rv nest	14	ACTGGACATTAGGCACCC
cmkC up fw	15	AGAACTCACTTGTCGTCCTC
cmkC up rv	16	CCAGCGCCTGCACCAGCTCCCGGACTCCGACTGAGCTTGG
cmkC dw fw	17	CAGTGCCTCCTCTCAGACAGTAAATAGGGCAACCAAAGAG
cmkC dw rv	18	TGTTGAGACTTTGACGGC
cmkC up fw nest	19	GCATCATCGTCTGTTGAC
cmkC dw rv nest	20	CTACATATCTCCCCGACGT
CaM left	21	GGCCAGAAAGATGAAGGACA
CaM right	22	CAGCAGCGGAGATGAAACC
cmkA left	23	TCAACAGAGAACAGCAACACACA
cmkA right	24	ACCTCACGGAAGATAGCACCTC
cmkB left	25	TACCGTCCGTGCCATCAA
cmkB right	26	CATCTTCATTCTCCCGCTCAC
cmkC left	27	ATGCCTCCACACCTTCGTTT
cmkC right	28	GACCGAGCATTGAGAGTTTCCT
actA left	29	GTCCTACGAACTGCCTGATGG
actA right	30	AAGAACGCTGGGCTGGAA

Video legends

Video 1. Conduction of calcium signal in the cut mycelia along the parallel direction to the hyphal growth, see Fig. 1A. The latter half is zoom in of former half. The calcium signals are shown in color boxes.

Video 2. Conduction of calcium signal in the cut mycelia vertically to the hyphal growth direction. The latter half, in the box area 800-1400 μm from the cut site (Fig. 1I, J), two waves of calcium signals.

Video 3. Conduction of calcium signal in the cut mycelia of 7- and 14-days cultured colonies (Fig. 1M).

Video 4. Decentralized stress response of mycelia by cut (Fig. 1N), drop of EtOH (Fig. S2I) and drop of NaCl (Fig. S2J).

Video 5. Conduction of calcium signal in the mycelia with a drop of EtOH (Fig. 2A), blinking of calcium signal at the hyphae in the color box area (Fig. 2E).

Video 6. Conduction of calcium signal in the mycelia with a drop of NaCl (Fig. 2H). The latter half, spread of calcium signals at the hyphae (Fig. 2L).

Video 7. Conduction of calcium signal in the mycelia of 7-days cultured colony with a drop of EtOH or NaCl (Fig. S2H).

Video 8. Conduction of calcium signal through hyphae stimulated by pointed laser irradiation. The elapsed time is given in min:sec.

Video 9. Conduction of calcium signal at the middle of the hypha. The latter half, conduction of calcium signal through the septa.

Video 10. Loss of F-actin signal (GFP-TpmA) at the tip by laser point irradiation, reconstruction of actin cytoskeleton and initiation of new hyphal growth. The latter half, loss of secretory vesicle signal (BglA-GFP) at the tip by laser point irradiation, reconstruction of growth sites and initiation of new hyphal growth.

Video 11. GFP-labeled microtubules and CaM-RFP. Total 140 s. Scale bar: 2 μm.
